# Acanthoic acid

**DOI:** 10.1107/S1600536810019483

**Published:** 2010-06-05

**Authors:** Sunisa Suwancharoen, Wantanee Tommeurd, Chuttree Phurat, Nongnuj Muangsin, Surachai Pornpakakul

**Affiliations:** a Research Centre of Bioorganic Chemistry, Department of Chemistry, Faculty of Science, Chulalongkorn University, Bangkok 10330, Thailand

## Abstract

The title compound [systematic name: (1*R*,4a*R*,7*S*,8a*S*,10a*S*)-1,4a,7-trimethyl-7-vinyl-1,2,3,4,4a,6,7,8,8a,9,10,10a-dodeca­hydro­phenanthrene-1-carb­oxy­lic acid], C_20_H_30_O_2_, is a pimarane-type diterpene extracted from *Croton oblongifolius*. There are two independent mol­ecules in the asymmetric unit. In both of these, the six-membered rings *A*, *B* and *C* adopt chair, boat and half-chair conformations, respectively. Rings *A* and *B* are *trans*-fused. The two mol­ecules in the asymmetric unit form O—H⋯O hydrogen-bonded *R*_2_^2^(8) dimers. The absolute configuration was assigned on the basis of the published literature on analogous structures.

## Related literature

For background to the structure of acanthoic acid, see: Kim *et al.* (1998 ▶); Ling *et al.* (2001 ▶); Suh *et al.* (2001 ▶). For the related absolute configuration, see: Ling *et al.* (2000 ▶). For puckering parameters, see: Cremer & Pople (1975 ▶).
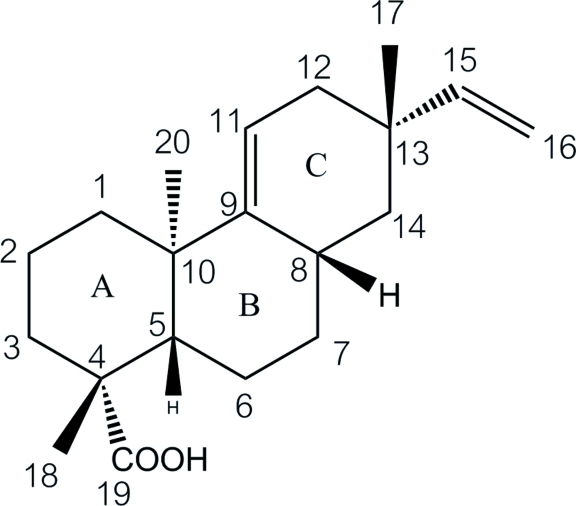


## Experimental

### 

#### Crystal data

C_20_H_30_O_2_
*M* *_r_* = 302.44
Tetragonal, 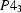

*a* = 12.8697 (16) Å
*c* = 21.768 (2) Å
*V* = 3605.5 (7) Å^3^
*Z* = 8
Mo *K*α radiation
μ = 0.07 mm^−1^
*T* = 100 K
0.40 × 0.20 × 0.02 mm

#### Data collection

Bruker SMART APEXII CCD area-detector diffractometer
21616 measured reflections
4824 independent reflections
3830 reflections with *I* > 2σ(*I*)
*R*_int_ = 0.050

#### Refinement

*R*[*F* ^2^ > 2σ(*F* ^2^)] = 0.056
*wR*(*F* ^2^) = 0.158
*S* = 1.02
4824 reflections
405 parameters
1 restraint
H-atom parameters constrained
Δρ_max_ = 0.37 e Å^−3^
Δρ_min_ = −0.29 e Å^−3^

### 

Data collection: *APEX2* (Bruker, 2008 ▶); cell refinement: *SAINT* (Bruker, 2008 ▶); data reduction: *SAINT*; program(s) used to solve structure: *SHELXS97* (Sheldrick, 2008 ▶); program(s) used to refine structure: *SHELXL97* (Sheldrick, 2008 ▶); molecular graphics: *ORTEP-3* (Farrugia, 1997 ▶); software used to prepare material for publication: *publCIF* (Westrip, 2010 ▶).

## Supplementary Material

Crystal structure: contains datablocks global, I. DOI: 10.1107/S1600536810019483/fj2300sup1.cif

Structure factors: contains datablocks I. DOI: 10.1107/S1600536810019483/fj2300Isup2.hkl

Additional supplementary materials:  crystallographic information; 3D view; checkCIF report

## Figures and Tables

**Table 1:** Hydrogen-bond geometry (Å, °)

*D*—H⋯*A*	*D*—H	H⋯*A*	*D*⋯*A*	*D*—H⋯*A*
O2′—H2′⋯O1	0.82	1.87	2.687 (3)	177
O2—H2⋯O1′	0.82	1.83	2.649 (3)	175

**Fig. 1.**
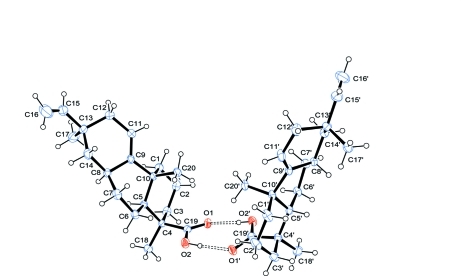
The asymmetric unit of the title compound showing two independent molecules. Displacement ellipsoids for non-H atoms are drawn at the 30% probability level. Hydrogen bonds are shown as dashed lines.

**Fig. 2.**
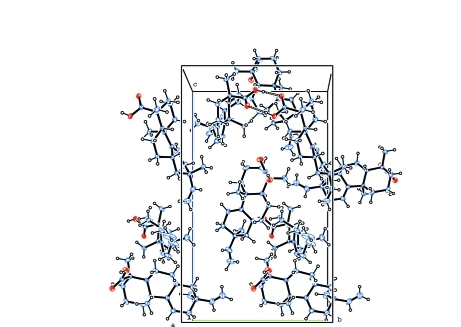
The crystal structure of the title compound viewed along the a-axis.

## supplementary crystallographic information

### Comment

Acanthoic acid is a pimarane-type diterpene. It was first isolated from root bark of *Acanthopanax koreanum* Nakai (Araliaceae) (Kim *et al.*, 1998) which has been used for treatment of neuralgia, hypertension, rheumatism and diabetes (Ling *et al.*, 2001). This natural product exhibits anti-inflammatory activity (Suh *et al.*, 2001). In this work, acanthoic acid was isolated in high yield from stem bark of *Croton oblongifolius* from Ratchaburi Province, Thailand.

There are two independent molecules in the asymmetric unit. In both independent molecules, the six memberred rings A, B and C adopts a chair, boat and half-chair conformations, respectively with the puckering parameters: Q = 0.546 Å, θ = 179.5° and φ = -107.0° for A, Q = 0.766 Å, θ = 89.9° and φ = -73.3° for B and Q = 0.493 Å, θ = 128.4° and φ = 35.2° for C. Rings A/B is *trans*-fused. The ethylene group substituted at C13 is in an equatorial position. The two molecules in the asymmetric unit form O—H···O hydrogen-bonded *R*_2_^2^(8) dimers. The absolute configuration was assigned by comparison with the crystal structure of *p*-bromobenzoate ester-acanthoic derivative (Ling *et al.*, 2000).

### Experimental

Dried powder of stem bark of *Croton oblongifolius* Roxb. (5.23 kg) from Ratchaburi province was extracted with hexane (4Lx5). The hexane crude extract was obtained as viscous yellow brown oil. This crude extract was purified by quick column chromatography on silica gel using a mixture of hexane and ethyl acetate (100:0-0:100). Fractions with similar components were combined according to TLC profile. The combined fraction eluted with a 7:3 mixture of hexane and ethyl acetate was crystallized in hexane and ethyl acetate to give colourless crystals (5.5% yield).

mp. 140-142^o^C; [a]^25^_D_ -36.1 (*c *= 0.42, benzene); ^1^H-NMR (400 MHz, CDCl_3_) *d* 5.81 (dd, 1H, *J*=10.6, 17.4 Hz, H-15), 5.39 (m, 1H, H-11), 4.93 (dd, 1H, *J*=1.2, 17.4 Hz, H-16* trans*), 4.86 (dd, 1H, *J*=1.2, 10.6 Hz, H-16 *cis*), 2.31 (m, 1H, H-8), 2.21 (m, 1H, H-2 b), 2.15 (m, 1H, H-3a), 2.01 (m, 1H, H-12a), 1.93 (m, 1H, H-2a), 1.89 (m, 1H, H-6 b), 1.81 (m, 1H, H-1a), 1.77 (m, 1H, H-12 b), 1.73 (m, 1H, H-7a), 1.66 (dd, 1H, *J*=6.2, 13.0 Hz, H-5), 1.48 (m, 1H, H-6a), 1.45 (m, 1H, H-14a), 1.28 (m, 1H, H-1 b), 1.25 (s, 3H, H-18), 1.21 (m, 1H, H-7 b), 1.05 (m, 1H, H-3 b), 1.03 (m, 1H, H-14 b), 0.99 (s, 3H, H-20), 0.96 (s, 3H, H-17); ^13^C NMR (100 MHz, CDCl_3_) *d* 184.60 (C-19), 150.23 (C-15), 149.85 (C-9), 116.59 (C-11), 109.16 (C-16), 47.99 (C-5), 44.21 (C-4), 41.92 (C-1), 41.80 (C-14), 38.43 (C-10), 38.08 (C-3), 37.47 (C-12), 34.86 (C-13), 28.67 (C-8), 28.56 (C-18), 27.76 (C-7), 22.40 (C-20), 22.17 (C-17), 20.34 (C-6), 18.91 (C-2)

### Refinement

Non-H atoms were refined anisotropically. H atoms were treated as riding atoms with distances C—H = 0.96 Å (CH_3_), 0.97 Å (CH_2_), 0.93 Å (CH), and *U*iso(H) = 1.20 *U*eq(C) for methylene and aromatic, 1.50 *U*eq(C) for methyl. The absolute structure could not be determined from the X-ray analysis, but it is known from earlier work on related compounds (Ling *et al.*, 2000). In the absence of significant anomalous scattering effects, 3,697 Friedel pairs were therefore merged before the final refinement.

### Crystal data

**Table:**

C_20_H_30_O_2_	*D*_x_ = 1.114 Mg m^−^^3^
*M**_r_* = 302.44	Mo *K*α radiation, λ = 0.71073 Å
Tetragonal, *P*4_3_	Cell parameters from 9639 reflections
Hall symbol: P 4cw	θ = 1.6–30.2°
*a* = 12.8697 (16) Å	µ = 0.07 mm^−^^1^
*c* = 21.768 (2) Å	*T* = 100 K
*V* = 3605.5 (7) Å^3^	Needle, colourless
*Z* = 8	0.40 × 0.20 × 0.02 mm
*F*(000) = 1328	

### Data collection

**Table:**

Bruker SMART APEXII CCD area-detector diffractometer	3830 reflections with *I* > 2σ(*I*)
Radiation source: Mo	*R*_int_ = 0.050
graphite	θ_max_ = 30.2°, θ_min_ = 1.6°
φ and ω scans	*h* = −16→16
21616 measured reflections	*k* = −17→17
4824 independent reflections	*l* = −25→28

### Refinement

**Table:**

Refinement on *F*^2^	1 restraint
Least-squares matrix: full	H-atom parameters constrained
*R*[*F*^2^ > 2σ(*F*^2^)] = 0.056	*w* = 1/[σ^2^(*F*_o_^2^) + (0.0853*P*)^2^ + 1.0669*P*] where *P* = (*F*_o_^2^ + 2*F*_c_^2^)/3
*wR*(*F*^2^) = 0.158	(Δ/σ)_max_ = 0.002
*S* = 1.02	Δρ_max_ = 0.37 e Å^−^^3^
4824 reflections	Δρ_min_ = −0.29 e Å^−^^3^
405 parameters	

### Special details

**Table:**

Geometry. All esds (except the esd in the dihedral angle between two l.s. planes) are estimated using the full covariance matrix. The cell esds are taken into account individually in the estimation of esds in distances, angles and torsion angles; correlations between esds in cell parameters are only used when they are defined by crystal symmetry. An approximate (isotropic) treatment of cell esds is used for estimating esds involving l.s. planes.

### Fractional atomic coordinates and isotropic or equivalent isotropic displacement parameters (Å^2^)

**Table:**

	*x*	*y*	*z*	*U*_iso_*/*U*_eq_	
C1	0.2020 (3)	0.6538 (3)	0.07071 (16)	0.0369 (7)	
H1A	0.133	0.6753	0.0833	0.044*	
H1B	0.208	0.6659	0.0269	0.044*	
C2	0.2143 (3)	0.5365 (3)	0.08320 (18)	0.0395 (8)	
H2A	0.1582	0.4991	0.0633	0.047*	
H2B	0.2793	0.5125	0.0657	0.047*	
C3	0.2130 (3)	0.5131 (3)	0.15123 (18)	0.0389 (7)	
H3A	0.2246	0.4393	0.157	0.047*	
H3B	0.1446	0.5293	0.1673	0.047*	
C4	0.2946 (2)	0.5735 (2)	0.18865 (14)	0.0283 (6)	
C5	0.2819 (2)	0.6927 (2)	0.17370 (13)	0.0257 (6)	
H5	0.2117	0.7103	0.1876	0.031*	
C6	0.3540 (2)	0.7641 (2)	0.21113 (15)	0.0332 (6)	
H6A	0.4256	0.7438	0.2041	0.04*	
H6B	0.3393	0.7553	0.2545	0.04*	
C7	0.3407 (3)	0.8792 (3)	0.19396 (17)	0.0384 (7)	
H7A	0.3387	0.9204	0.2313	0.046*	
H7B	0.4005	0.9015	0.1703	0.046*	
C8	0.2417 (2)	0.9002 (2)	0.15664 (15)	0.0309 (6)	
H8	0.182	0.8755	0.1805	0.037*	
C9	0.2483 (2)	0.8366 (2)	0.09816 (14)	0.0307 (6)	
C10	0.2833 (2)	0.7217 (2)	0.10447 (13)	0.0261 (6)	
C11	0.2242 (3)	0.8779 (3)	0.04395 (17)	0.0439 (8)	
H11	0.2322	0.8361	0.0094	0.053*	
C12	0.1851 (4)	0.9870 (3)	0.03404 (18)	0.0535 (10)	
H12A	0.1287	0.9856	0.0045	0.064*	
H12B	0.2406	1.0289	0.0169	0.064*	
C13	0.1463 (3)	1.0377 (3)	0.09429 (18)	0.0481 (9)	
C14	0.2265 (3)	1.0152 (3)	0.14356 (18)	0.0436 (8)	
H14A	0.2055	1.0496	0.1812	0.052*	
H14B	0.2925	1.0447	0.131	0.052*	
C15	0.1282 (5)	1.1516 (4)	0.0829 (2)	0.0853 (19)	
H15	0.0933	1.1636	0.0461	0.102*	
C16	0.1472 (8)	1.2286 (4)	0.1089 (3)	0.123 (3)	
H16A	0.182	1.2263	0.1463	0.147*	
H16B	0.1275	1.2921	0.0922	0.147*	
C17	0.0409 (3)	0.9895 (4)	0.1120 (2)	0.0610 (12)	
H17A	0.0484	0.9156	0.1159	0.092*	
H17B	−0.0093	1.0047	0.0806	0.092*	
H17C	0.018	1.0181	0.1504	0.092*	
C18	0.2726 (3)	0.5543 (3)	0.25738 (17)	0.0429 (8)	
H18A	0.2051	0.5811	0.2676	0.064*	
H18B	0.3243	0.5888	0.2817	0.064*	
H18C	0.2745	0.481	0.2656	0.064*	
C19	0.4032 (2)	0.5295 (2)	0.17572 (14)	0.0312 (6)	
C20	0.3915 (2)	0.7082 (2)	0.07483 (14)	0.0332 (7)	
H20A	0.3909	0.7371	0.0342	0.05*	
H20B	0.4083	0.6356	0.0726	0.05*	
H20C	0.4427	0.7434	0.0993	0.05*	
O1	0.42045 (17)	0.46242 (17)	0.13726 (11)	0.0352 (5)	
O2	0.47604 (18)	0.5668 (2)	0.21170 (12)	0.0437 (6)	
H2	0.532	0.5404	0.2027	0.066*	
C1'	0.8991 (3)	0.6408 (3)	0.09529 (17)	0.0415 (8)	
H1'1	0.9703	0.6216	0.086	0.05*	
H1'2	0.8959	0.716	0.0969	0.05*	
C2'	0.8713 (3)	0.5981 (3)	0.15830 (16)	0.0468 (9)	
H2'1	0.9222	0.6216	0.1881	0.056*	
H2'2	0.8039	0.6248	0.1706	0.056*	
C3'	0.8682 (3)	0.4805 (3)	0.15808 (16)	0.0429 (8)	
H3'1	0.8463	0.4564	0.1983	0.051*	
H3'2	0.9377	0.4542	0.1507	0.051*	
C4'	0.7945 (2)	0.4360 (2)	0.10933 (15)	0.0322 (6)	
C5'	0.8256 (2)	0.4816 (2)	0.04527 (14)	0.0271 (6)	
H5'	0.8981	0.4603	0.0397	0.033*	
C6'	0.7679 (2)	0.4328 (2)	−0.00971 (15)	0.0316 (6)	
H6'1	0.7876	0.3603	−0.0131	0.038*	
H6'2	0.6937	0.4357	−0.0021	0.038*	
C7'	0.7919 (2)	0.4880 (2)	−0.07080 (14)	0.0299 (6)	
H7'1	0.7984	0.4365	−0.1031	0.036*	
H7'2	0.7342	0.5333	−0.0813	0.036*	
C8'	0.8922 (2)	0.5526 (2)	−0.06797 (13)	0.0257 (6)	
H8'	0.9494	0.5068	−0.0557	0.031*	
C9'	0.8790 (2)	0.6354 (2)	−0.01921 (15)	0.0301 (6)	
C10'	0.8288 (2)	0.6029 (2)	0.04225 (14)	0.0285 (6)	
C11'	0.9096 (3)	0.7328 (2)	−0.02861 (17)	0.0402 (8)	
H11'	0.8987	0.7801	0.003	0.048*	
C12'	0.9605 (3)	0.7729 (3)	−0.08629 (19)	0.0435 (8)	
H12C	1.018	0.8177	−0.0751	0.052*	
H12D	0.9106	0.8144	−0.109	0.052*	
C13'	1.0012 (2)	0.6844 (3)	−0.12842 (16)	0.0355 (7)	
C14'	0.9179 (2)	0.5993 (2)	−0.13061 (14)	0.0332 (6)	
H14C	0.8549	0.6283	−0.1481	0.04*	
H14D	0.9414	0.5442	−0.1576	0.04*	
C15'	1.0229 (3)	0.7320 (3)	−0.1906 (2)	0.0536 (10)	
H15'	1.0743	0.7829	−0.1908	0.064*	
C16'	0.9827 (4)	0.7138 (5)	−0.2418 (2)	0.0762 (15)	
H16C	0.9307	0.664	−0.2453	0.091*	
H16D	1.0047	0.7502	−0.2763	0.091*	
C17'	1.1037 (2)	0.6415 (3)	−0.10289 (17)	0.0401 (8)	
H17D	1.1267	0.5847	−0.128	0.06*	
H17E	1.0932	0.6176	−0.0616	0.06*	
H17F	1.1553	0.6954	−0.1031	0.06*	
C18'	0.8066 (3)	0.3167 (3)	0.10861 (19)	0.0447 (8)	
H18D	0.7936	0.2897	0.149	0.067*	
H18E	0.876	0.2989	0.0963	0.067*	
H18F	0.7579	0.2872	0.0801	0.067*	
C19'	0.6819 (2)	0.4561 (2)	0.12987 (15)	0.0318 (6)	
C20'	0.7191 (2)	0.6510 (2)	0.04652 (16)	0.0338 (7)	
H20D	0.723	0.7242	0.0383	0.051*	
H20E	0.6916	0.6401	0.087	0.051*	
H20F	0.6743	0.6185	0.0169	0.051*	
O1'	0.66053 (17)	0.4932 (2)	0.17988 (11)	0.0402 (5)	
O2'	0.61042 (16)	0.42401 (18)	0.09132 (11)	0.0362 (5)	
H2'	0.5527	0.4336	0.1063	0.054*	

### Atomic displacement parameters (Å^2^)

**Table:**

	*U*^11^	*U*^22^	*U*^33^	*U*^12^	*U*^13^	*U*^23^
C1	0.0352 (16)	0.0399 (17)	0.0356 (17)	0.0035 (13)	−0.0104 (13)	−0.0085 (13)
C2	0.0300 (15)	0.0364 (17)	0.052 (2)	−0.0012 (13)	−0.0083 (14)	−0.0164 (15)
C3	0.0293 (15)	0.0311 (15)	0.056 (2)	−0.0040 (12)	0.0048 (14)	−0.0024 (15)
C4	0.0268 (13)	0.0308 (14)	0.0273 (14)	0.0015 (11)	0.0060 (11)	0.0024 (11)
C5	0.0238 (12)	0.0294 (14)	0.0241 (14)	0.0008 (10)	0.0007 (10)	−0.0004 (11)
C6	0.0321 (15)	0.0382 (16)	0.0292 (15)	−0.0013 (12)	−0.0035 (12)	−0.0085 (13)
C7	0.0365 (16)	0.0379 (17)	0.0408 (19)	−0.0064 (13)	−0.0014 (14)	−0.0107 (14)
C8	0.0306 (14)	0.0302 (14)	0.0320 (15)	−0.0015 (11)	0.0090 (12)	−0.0022 (12)
C9	0.0304 (14)	0.0284 (14)	0.0333 (16)	0.0010 (11)	0.0095 (12)	0.0000 (12)
C10	0.0267 (13)	0.0283 (13)	0.0232 (14)	0.0010 (10)	0.0013 (10)	−0.0035 (11)
C11	0.056 (2)	0.0450 (19)	0.0308 (17)	0.0130 (16)	0.0074 (15)	0.0021 (14)
C12	0.070 (3)	0.050 (2)	0.041 (2)	0.0220 (19)	0.0139 (18)	0.0119 (17)
C13	0.064 (2)	0.0360 (17)	0.044 (2)	0.0177 (16)	0.0181 (18)	0.0112 (15)
C14	0.054 (2)	0.0311 (16)	0.046 (2)	0.0001 (14)	0.0140 (16)	−0.0039 (14)
C15	0.152 (5)	0.046 (3)	0.057 (3)	0.037 (3)	0.043 (3)	0.019 (2)
C16	0.259 (10)	0.047 (3)	0.063 (4)	0.002 (4)	0.052 (5)	0.001 (3)
C17	0.049 (2)	0.066 (3)	0.068 (3)	0.019 (2)	0.007 (2)	0.017 (2)
C18	0.0443 (19)	0.0463 (19)	0.0381 (19)	0.0087 (15)	0.0151 (15)	0.0136 (15)
C19	0.0302 (14)	0.0334 (15)	0.0301 (16)	0.0031 (12)	0.0049 (12)	0.0063 (12)
C20	0.0337 (15)	0.0368 (16)	0.0290 (16)	0.0009 (12)	0.0090 (12)	−0.0002 (12)
O1	0.0297 (11)	0.0339 (11)	0.0420 (13)	0.0043 (9)	0.0057 (9)	−0.0020 (9)
O2	0.0306 (11)	0.0593 (16)	0.0413 (14)	0.0109 (11)	−0.0038 (10)	−0.0125 (12)
C1'	0.0365 (17)	0.0484 (19)	0.0395 (18)	−0.0164 (14)	0.0051 (14)	−0.0145 (15)
C2'	0.0376 (18)	0.070 (2)	0.0332 (18)	−0.0149 (17)	0.0003 (14)	−0.0156 (17)
C3'	0.0282 (15)	0.070 (2)	0.0309 (17)	−0.0012 (15)	0.0022 (13)	−0.0002 (16)
C4'	0.0258 (14)	0.0394 (16)	0.0312 (16)	0.0013 (12)	0.0038 (12)	0.0000 (12)
C5'	0.0217 (12)	0.0287 (13)	0.0309 (15)	−0.0003 (10)	0.0043 (11)	−0.0007 (11)
C6'	0.0336 (15)	0.0293 (14)	0.0319 (16)	−0.0072 (12)	0.0048 (12)	−0.0071 (12)
C7'	0.0295 (14)	0.0318 (14)	0.0284 (15)	−0.0045 (11)	−0.0005 (11)	−0.0061 (12)
C8'	0.0259 (13)	0.0252 (13)	0.0261 (14)	0.0008 (10)	0.0033 (11)	−0.0027 (11)
C9'	0.0251 (13)	0.0291 (14)	0.0363 (16)	−0.0030 (11)	0.0088 (12)	−0.0072 (12)
C10'	0.0255 (13)	0.0261 (13)	0.0337 (16)	−0.0048 (10)	0.0063 (11)	−0.0087 (11)
C11'	0.0442 (18)	0.0266 (15)	0.050 (2)	−0.0019 (13)	0.0177 (15)	−0.0090 (14)
C12'	0.0411 (18)	0.0291 (16)	0.060 (2)	−0.0036 (13)	0.0171 (16)	0.0034 (15)
C13'	0.0319 (15)	0.0380 (16)	0.0366 (17)	−0.0001 (12)	0.0098 (13)	0.0089 (13)
C14'	0.0330 (15)	0.0371 (16)	0.0296 (15)	0.0026 (12)	0.0022 (12)	0.0000 (12)
C15'	0.045 (2)	0.063 (2)	0.053 (3)	0.0037 (17)	0.0147 (18)	0.022 (2)
C16'	0.079 (3)	0.111 (4)	0.039 (3)	0.004 (3)	0.008 (2)	0.013 (2)
C17'	0.0322 (16)	0.0484 (19)	0.0398 (19)	−0.0012 (13)	0.0072 (14)	0.0047 (15)
C18'	0.0447 (19)	0.0415 (18)	0.048 (2)	0.0055 (15)	0.0097 (16)	0.0135 (16)
C19'	0.0259 (14)	0.0368 (16)	0.0328 (16)	−0.0006 (11)	0.0042 (12)	0.0027 (12)
C20'	0.0332 (15)	0.0296 (14)	0.0387 (17)	0.0017 (12)	0.0126 (13)	−0.0055 (13)
O1'	0.0287 (11)	0.0606 (15)	0.0312 (12)	0.0039 (10)	0.0038 (9)	−0.0057 (11)
O2'	0.0251 (10)	0.0459 (13)	0.0374 (12)	−0.0053 (9)	0.0070 (9)	−0.0049 (10)

### Geometric parameters (Å, °)

**Table:**

C1—C2	1.542 (5)	C1'—C2'	1.520 (5)
C1—C10	1.548 (4)	C1'—C10'	1.545 (4)
C1—H1A	0.97	C1'—H1'1	0.97
C1—H1B	0.97	C1'—H1'2	0.97
C2—C3	1.511 (6)	C2'—C3'	1.514 (6)
C2—H2A	0.97	C2'—H2'1	0.97
C2—H2B	0.97	C2'—H2'2	0.97
C3—C4	1.540 (4)	C3'—C4'	1.534 (5)
C3—H3A	0.97	C3'—H3'1	0.97
C3—H3B	0.97	C3'—H3'2	0.97
C4—C19	1.534 (4)	C4'—C19'	1.538 (4)
C4—C18	1.543 (4)	C4'—C18'	1.544 (5)
C4—C5	1.577 (4)	C4'—C5'	1.565 (4)
C5—C6	1.539 (4)	C5'—C6'	1.542 (4)
C5—C10	1.553 (4)	C5'—C10'	1.563 (4)
C5—H5	0.98	C5'—H5'	0.98
C6—C7	1.537 (5)	C6'—C7'	1.539 (4)
C6—H6A	0.97	C6'—H6'1	0.97
C6—H6B	0.97	C6'—H6'2	0.97
C7—C8	1.535 (5)	C7'—C8'	1.536 (4)
C7—H7A	0.97	C7'—H7'1	0.97
C7—H7B	0.97	C7'—H7'2	0.97
C8—C9	1.515 (4)	C8'—C9'	1.514 (4)
C8—C14	1.520 (4)	C8'—C14'	1.526 (4)
C8—H8	0.98	C8'—H8'	0.98
C9—C11	1.331 (5)	C9'—C11'	1.329 (4)
C9—C10	1.552 (4)	C9'—C10'	1.544 (4)
C10—C20	1.545 (4)	C10'—C20'	1.545 (4)
C11—C12	1.507 (5)	C11'—C12'	1.507 (5)
C11—H11	0.93	C11'—H11'	0.93
C12—C13	1.548 (5)	C12'—C13'	1.553 (5)
C12—H12A	0.97	C12'—H12C	0.97
C12—H12B	0.97	C12'—H12D	0.97
C13—C15	1.505 (5)	C13'—C15'	1.511 (5)
C13—C14	1.516 (6)	C13'—C17'	1.534 (5)
C13—C17	1.540 (6)	C13'—C14'	1.534 (5)
C14—H14A	0.97	C14'—H14C	0.97
C14—H14B	0.97	C14'—H14D	0.97
C15—C16	1.167 (8)	C15'—C16'	1.251 (7)
C15—H15	0.93	C15'—H15'	0.93
C16—H16A	0.93	C16'—H16C	0.93
C16—H16B	0.93	C16'—H16D	0.93
C17—H17A	0.96	C17'—H17D	0.96
C17—H17B	0.96	C17'—H17E	0.96
C17—H17C	0.96	C17'—H17F	0.96
C18—H18A	0.96	C18'—H18D	0.96
C18—H18B	0.96	C18'—H18E	0.96
C18—H18C	0.96	C18'—H18F	0.96
C19—O1	1.223 (4)	C19'—O1'	1.220 (4)
C19—O2	1.313 (4)	C19'—O2'	1.312 (4)
C20—H20A	0.96	C20'—H20D	0.96
C20—H20B	0.96	C20'—H20E	0.96
C20—H20C	0.96	C20'—H20F	0.96
O2—H2	0.82	O2'—H2'	0.82
			
C2—C1—C10	113.6 (3)	C2'—C1'—C10'	115.0 (3)
C2—C1—H1A	108.9	C2'—C1'—H1'1	108.5
C10—C1—H1A	108.9	C10'—C1'—H1'1	108.5
C2—C1—H1B	108.9	C2'—C1'—H1'2	108.5
C10—C1—H1B	108.9	C10'—C1'—H1'2	108.5
H1A—C1—H1B	107.7	H1'1—C1'—H1'2	107.5
C3—C2—C1	111.5 (3)	C3'—C2'—C1'	111.4 (3)
C3—C2—H2A	109.3	C3'—C2'—H2'1	109.4
C1—C2—H2A	109.3	C1'—C2'—H2'1	109.4
C3—C2—H2B	109.3	C3'—C2'—H2'2	109.4
C1—C2—H2B	109.3	C1'—C2'—H2'2	109.4
H2A—C2—H2B	108	H2'1—C2'—H2'2	108
C2—C3—C4	114.2 (3)	C2'—C3'—C4'	113.1 (3)
C2—C3—H3A	108.7	C2'—C3'—H3'1	109
C4—C3—H3A	108.7	C4'—C3'—H3'1	109
C2—C3—H3B	108.7	C2'—C3'—H3'2	109
C4—C3—H3B	108.7	C4'—C3'—H3'2	109
H3A—C3—H3B	107.6	H3'1—C3'—H3'2	107.8
C19—C4—C3	109.8 (3)	C3'—C4'—C19'	108.6 (3)
C19—C4—C18	106.6 (2)	C3'—C4'—C18'	108.4 (3)
C3—C4—C18	107.9 (3)	C19'—C4'—C18'	105.4 (3)
C19—C4—C5	114.6 (2)	C3'—C4'—C5'	108.6 (2)
C3—C4—C5	108.1 (2)	C19'—C4'—C5'	115.9 (3)
C18—C4—C5	109.7 (2)	C18'—C4'—C5'	109.8 (3)
C6—C5—C10	111.3 (2)	C6'—C5'—C10'	112.8 (2)
C6—C5—C4	114.2 (2)	C6'—C5'—C4'	114.6 (2)
C10—C5—C4	115.6 (2)	C10'—C5'—C4'	114.8 (2)
C6—C5—H5	104.8	C6'—C5'—H5'	104.4
C10—C5—H5	104.8	C10'—C5'—H5'	104.4
C4—C5—H5	104.8	C4'—C5'—H5'	104.4
C5—C6—C7	112.3 (2)	C7'—C6'—C5'	112.7 (2)
C5—C6—H6A	109.1	C7'—C6'—H6'1	109
C7—C6—H6A	109.1	C5'—C6'—H6'1	109
C5—C6—H6B	109.1	C7'—C6'—H6'2	109
C7—C6—H6B	109.1	C5'—C6'—H6'2	109
H6A—C6—H6B	107.9	H6'1—C6'—H6'2	107.8
C8—C7—C6	113.0 (2)	C8'—C7'—C6'	112.5 (2)
C8—C7—H7A	109	C8'—C7'—H7'1	109.1
C6—C7—H7A	109	C6'—C7'—H7'1	109.1
C8—C7—H7B	109	C8'—C7'—H7'2	109.1
C6—C7—H7B	109	C6'—C7'—H7'2	109.1
H7A—C7—H7B	107.8	H7'1—C7'—H7'2	107.8
C9—C8—C14	112.0 (3)	C9'—C8'—C14'	111.9 (2)
C9—C8—C7	107.7 (3)	C9'—C8'—C7'	108.4 (2)
C14—C8—C7	112.1 (3)	C14'—C8'—C7'	111.1 (2)
C9—C8—H8	108.3	C9'—C8'—H8'	108.5
C14—C8—H8	108.3	C14'—C8'—H8'	108.5
C7—C8—H8	108.3	C7'—C8'—H8'	108.5
C11—C9—C8	121.1 (3)	C11'—C9'—C8'	121.5 (3)
C11—C9—C10	121.8 (3)	C11'—C9'—C10'	120.9 (3)
C8—C9—C10	117.1 (3)	C8'—C9'—C10'	117.6 (2)
C20—C10—C1	110.3 (3)	C20'—C10'—C9'	109.0 (3)
C20—C10—C9	109.4 (2)	C20'—C10'—C1'	111.3 (2)
C1—C10—C9	107.5 (2)	C9'—C10'—C1'	108.5 (2)
C20—C10—C5	112.8 (2)	C20'—C10'—C5'	111.9 (2)
C1—C10—C5	108.5 (2)	C9'—C10'—C5'	108.6 (2)
C9—C10—C5	108.1 (2)	C1'—C10'—C5'	107.4 (3)
C9—C11—C12	125.2 (3)	C9'—C11'—C12'	125.5 (3)
C9—C11—H11	117.4	C9'—C11'—H11'	117.3
C12—C11—H11	117.4	C12'—C11'—H11'	117.3
C11—C12—C13	112.3 (3)	C11'—C12'—C13'	112.8 (3)
C11—C12—H12A	109.1	C11'—C12'—H12C	109
C13—C12—H12A	109.1	C13'—C12'—H12C	109
C11—C12—H12B	109.1	C11'—C12'—H12D	109
C13—C12—H12B	109.1	C13'—C12'—H12D	109
H12A—C12—H12B	107.9	H12C—C12'—H12D	107.8
C15—C13—C14	114.1 (4)	C15'—C13'—C17'	108.1 (3)
C15—C13—C17	107.3 (4)	C15'—C13'—C14'	113.0 (3)
C14—C13—C17	110.2 (3)	C17'—C13'—C14'	110.8 (3)
C15—C13—C12	108.7 (3)	C15'—C13'—C12'	107.1 (3)
C14—C13—C12	107.4 (3)	C17'—C13'—C12'	109.9 (3)
C17—C13—C12	109.0 (4)	C14'—C13'—C12'	107.8 (3)
C13—C14—C8	114.0 (3)	C8'—C14'—C13'	113.9 (3)
C13—C14—H14A	108.8	C8'—C14'—H14C	108.8
C8—C14—H14A	108.8	C13'—C14'—H14C	108.8
C13—C14—H14B	108.8	C8'—C14'—H14D	108.8
C8—C14—H14B	108.8	C13'—C14'—H14D	108.8
H14A—C14—H14B	107.7	H14C—C14'—H14D	107.7
C16—C15—C13	135.5 (7)	C16'—C15'—C13'	130.2 (4)
C16—C15—H15	112.2	C16'—C15'—H15'	114.9
C13—C15—H15	112.2	C13'—C15'—H15'	114.9
C15—C16—H16A	120	C15'—C16'—H16C	120
C15—C16—H16B	120	C15'—C16'—H16D	120
H16A—C16—H16B	120	H16C—C16'—H16D	120
C13—C17—H17A	109.5	C13'—C17'—H17D	109.5
C13—C17—H17B	109.5	C13'—C17'—H17E	109.5
H17A—C17—H17B	109.5	H17D—C17'—H17E	109.5
C13—C17—H17C	109.5	C13'—C17'—H17F	109.5
H17A—C17—H17C	109.5	H17D—C17'—H17F	109.5
H17B—C17—H17C	109.5	H17E—C17'—H17F	109.5
C4—C18—H18A	109.5	C4'—C18'—H18D	109.5
C4—C18—H18B	109.5	C4'—C18'—H18E	109.5
H18A—C18—H18B	109.5	H18D—C18'—H18E	109.5
C4—C18—H18C	109.5	C4'—C18'—H18F	109.5
H18A—C18—H18C	109.5	H18D—C18'—H18F	109.5
H18B—C18—H18C	109.5	H18E—C18'—H18F	109.5
O1—C19—O2	122.5 (3)	O1'—C19'—O2'	122.4 (3)
O1—C19—C4	123.5 (3)	O1'—C19'—C4'	122.5 (3)
O2—C19—C4	114.0 (3)	O2'—C19'—C4'	114.9 (3)
C10—C20—H20A	109.5	C10'—C20'—H20D	109.5
C10—C20—H20B	109.5	C10'—C20'—H20E	109.5
H20A—C20—H20B	109.5	H20D—C20'—H20E	109.5
C10—C20—H20C	109.5	C10'—C20'—H20F	109.5
H20A—C20—H20C	109.5	H20D—C20'—H20F	109.5
H20B—C20—H20C	109.5	H20E—C20'—H20F	109.5
C19—O2—H2	109.5	C19'—O2'—H2'	109.5
			
C10—C1—C2—C3	54.8 (4)	C10'—C1'—C2'—C3'	54.7 (4)
C1—C2—C3—C4	−55.4 (4)	C1'—C2'—C3'—C4'	−55.4 (4)
C2—C3—C4—C19	−72.7 (3)	C2'—C3'—C4'—C19'	−72.1 (3)
C2—C3—C4—C18	171.5 (3)	C2'—C3'—C4'—C18'	173.9 (3)
C2—C3—C4—C5	52.9 (3)	C2'—C3'—C4'—C5'	54.7 (3)
C19—C4—C5—C6	−61.0 (3)	C3'—C4'—C5'—C6'	172.2 (3)
C3—C4—C5—C6	176.2 (3)	C19'—C4'—C5'—C6'	−65.4 (3)
C18—C4—C5—C6	58.8 (3)	C18'—C4'—C5'—C6'	53.8 (3)
C19—C4—C5—C10	70.1 (3)	C3'—C4'—C5'—C10'	−55.0 (3)
C3—C4—C5—C10	−52.7 (3)	C19'—C4'—C5'—C10'	67.5 (3)
C18—C4—C5—C10	−170.1 (3)	C18'—C4'—C5'—C10'	−173.3 (3)
C10—C5—C6—C7	45.5 (3)	C10'—C5'—C6'—C7'	39.9 (3)
C4—C5—C6—C7	178.7 (2)	C4'—C5'—C6'—C7'	173.7 (2)
C5—C6—C7—C8	14.7 (4)	C5'—C6'—C7'—C8'	19.4 (4)
C6—C7—C8—C9	−59.8 (3)	C6'—C7'—C8'—C9'	−61.1 (3)
C6—C7—C8—C14	176.4 (3)	C6'—C7'—C8'—C14'	175.6 (2)
C14—C8—C9—C11	−11.1 (4)	C14'—C8'—C9'—C11'	−13.8 (4)
C7—C8—C9—C11	−134.9 (3)	C7'—C8'—C9'—C11'	−136.6 (3)
C14—C8—C9—C10	169.4 (3)	C14'—C8'—C9'—C10'	166.3 (3)
C7—C8—C9—C10	45.6 (3)	C7'—C8'—C9'—C10'	43.5 (3)
C2—C1—C10—C20	71.9 (4)	C11'—C9'—C10'—C20'	71.0 (4)
C2—C1—C10—C9	−169.0 (3)	C8'—C9'—C10'—C20'	−109.2 (3)
C2—C1—C10—C5	−52.2 (3)	C11'—C9'—C10'—C1'	−50.4 (4)
C11—C9—C10—C20	68.8 (4)	C8'—C9'—C10'—C1'	129.4 (3)
C8—C9—C10—C20	−111.7 (3)	C11'—C9'—C10'—C5'	−166.9 (3)
C11—C9—C10—C1	−51.0 (4)	C8'—C9'—C10'—C5'	13.0 (3)
C8—C9—C10—C1	128.5 (3)	C2'—C1'—C10'—C20'	70.8 (4)
C11—C9—C10—C5	−168.0 (3)	C2'—C1'—C10'—C9'	−169.3 (3)
C8—C9—C10—C5	11.6 (3)	C2'—C1'—C10'—C5'	−52.1 (4)
C6—C5—C10—C20	62.5 (3)	C6'—C5'—C10'—C20'	64.1 (3)
C4—C5—C10—C20	−70.0 (3)	C4'—C5'—C10'—C20'	−69.6 (3)
C6—C5—C10—C1	−174.9 (2)	C6'—C5'—C10'—C9'	−56.3 (3)
C4—C5—C10—C1	52.6 (3)	C4'—C5'—C10'—C9'	170.0 (2)
C6—C5—C10—C9	−58.7 (3)	C6'—C5'—C10'—C1'	−173.5 (2)
C4—C5—C10—C9	168.9 (2)	C4'—C5'—C10'—C1'	52.8 (3)
C8—C9—C11—C12	−2.4 (6)	C8'—C9'—C11'—C12'	−0.6 (6)
C10—C9—C11—C12	177.1 (3)	C10'—C9'—C11'—C12'	179.2 (3)
C9—C11—C12—C13	−16.0 (6)	C9'—C11'—C12'—C13'	−15.2 (5)
C11—C12—C13—C15	169.3 (4)	C11'—C12'—C13'—C15'	165.0 (3)
C11—C12—C13—C14	45.4 (5)	C11'—C12'—C13'—C17'	−77.8 (4)
C11—C12—C13—C17	−74.0 (4)	C11'—C12'—C13'—C14'	43.1 (4)
C15—C13—C14—C8	177.8 (3)	C9'—C8'—C14'—C13'	45.2 (3)
C17—C13—C14—C8	57.1 (4)	C7'—C8'—C14'—C13'	166.5 (3)
C12—C13—C14—C8	−61.6 (4)	C15'—C13'—C14'—C8'	−178.1 (3)
C9—C8—C14—C13	44.4 (4)	C17'—C13'—C14'—C8'	60.3 (4)
C7—C8—C14—C13	165.6 (3)	C12'—C13'—C14'—C8'	−60.0 (4)
C14—C13—C15—C16	−15.6 (10)	C17'—C13'—C15'—C16'	124.4 (5)
C17—C13—C15—C16	106.8 (9)	C14'—C13'—C15'—C16'	1.3 (6)
C12—C13—C15—C16	−135.4 (8)	C12'—C13'—C15'—C16'	−117.2 (5)
C3—C4—C19—O1	5.7 (4)	C3'—C4'—C19'—O1'	−6.0 (4)
C18—C4—C19—O1	122.2 (3)	C18'—C4'—C19'—O1'	110.0 (4)
C5—C4—C19—O1	−116.2 (3)	C5'—C4'—C19'—O1'	−128.4 (3)
C3—C4—C19—O2	−171.4 (3)	C3'—C4'—C19'—O2'	178.4 (3)
C18—C4—C19—O2	−54.8 (3)	C18'—C4'—C19'—O2'	−65.6 (3)
C5—C4—C19—O2	66.8 (3)	C5'—C4'—C19'—O2'	55.9 (4)

### Hydrogen-bond geometry (Å, °)

**Table:**

*D*—H···*A*	*D*—H	H···*A*	*D*···*A*	*D*—H···*A*
O2'—H2'···O1	0.82	1.87	2.687 (3)	177
O2—H2···O1'	0.82	1.83	2.649 (3)	175
